# Ebola a reality of modern Public Health; need for Surveillance, Preparedness and Response Training for Health Workers and other multidisciplinary teams: a case for Uganda

**DOI:** 10.11604/pamj.2015.20.404.6159

**Published:** 2015-04-23

**Authors:** William Bazeyo, James Bagonza, Ali Halage, Gildo Okure, Malimbo Mugagga, Robert Musoke, Mathias Tumwebaze, Suzan Tusiime, Steven Ssendagire, Immaculate Nabukenya, Steven Pande, Christine Aanyu, Samuel Etajak, Elizeus Rutebemberwa

**Affiliations:** 1 School of Public Health, Makerere University College of Health sciences; 2 Ministry of Health; 3 District Health Office, Kabarole District Local Government; 4 Medical Division, Uganda People's Defence Forces

**Keywords:** Ebola, surveillance, emergency, preparedness, response, training

## Abstract

**Introduction:**

West Africa is experiencing the largest ever reported Ebola outbreak. Over 20,000 people have been infected of which about 9000 have died. It is possible that lack of community understanding of the epidemic and lack of institutional memory and inexperienced health workers could have led to the rapid spread of the disease. In this paper, we share Uganda's experiences on how the capacity of health workers and other multidisciplinary teams can be improved in preparing and responding to Ebola outbreaks.

**Methods:**

Makerere University School of Public Health in collaboration with the Ministry of Health and the African Field Epidemiology Network (AFENET), trained health care workers and other multidisciplinary teams from six border districts of Uganda so as to increase their alertness and response capabilities towards Ebola. We used participatory training methods to impart knowledge and skills and guided participants to develop district epidemic response plans. Communities were sensitized about Ebola through mass media, IEC materials, and infection control and prevention materials were distributed in districts.

**Results:**

We trained 210 health workers and 120 other multidisciplinary team members on Ebola surveillance, preparedness and response. Evaluation results demonstrated a gain in knowledge and skills. Communities were sensitized about Ebola and Districts received person protective equipments and items for infection prevention. Epidemic Preparedness and Response plans were also developed.

**Conclusion:**

Training of multidisciplinary teams improves the country's preparedness, alertness and response capabilities in controlling Ebola. West African countries experiencing Ebola outbreaks could draw lessons from the Uganda experience to contain the outbreak.

## Introduction

Since 1976 when the Ebola virus was first identified, no Ebola Virus Disease (EVD) outbreak has been as large or geographically widespread or persistent as the current epidemic in West Africa. Worse still, this is the first time that EVD is affecting West Africa; the disease was previously limited to East and Central Africa [1, 2] With each passing day, news of the ongoing Ebola outbreak becomes more dire [3]. EVD is spreading across Guinea, Sierra Leone and Liberia at an unprecedented and exponential rate, with global health agencies predicting 10,000 new cases a week by the end of 2014 culminating in hundreds of thousands of affected people if response efforts are not rapidly strengthened [4, 5]. As of 29 October, there were 13,567 confirmed, probable, and suspected cases of EVD reported in eight affected countries- Guinea, Liberia, Mali, Sierra Leone, Spain, Nigeria, Senegal and the United States with 4,951 deaths [6]. The reported number of EVD cases exceeds that from all previous outbreaks combined [5, 7]

According to the United Nations Mission for Ebola Emergency Response (UNMEER) 4,199 cases and 1,023 deaths have occurred in Sierra Leone (24% Case fatality rate). However, the case fatality rate among healthcare workers (HCWs) in Sierra Leone is very high at 80% with 101 deaths out of 127 infected HCWs (as of 26 October 2014). This is worrying given the limited number of health care workers; there were only 100 doctors serving 6 million citizens prior to the EVD outbreak, translating into two doctors per 100,000 people [8, 9]. Weak health institutions, marred by the county's 11-year civil war coupled with low numbers of trained local HCWs have contributed to the country's inability to properly recognize, isolate and treat patients, bury their dead or contain the disease's spread [3, 7, 10]. The ongoing EVD outbreak has weakened the entire health system of the three most affected countries of Sierra Leone, Liberia and Guinea given that its fragile state prior to the onset of the outbreak [4, 11] noted that Sierra Leone's non-Ebola health system has collapsed at the height of the epidemic, and the university medical and health programs have been shuttered for months.

The International Health Regulations 2005 (IHR) require every country to develop its capacity to detect and respond to public health events of potential international concern. There is an immediate need to train health care professionals to provide critical care in Ebola Treatment Centres (ETCs), district and community level health centers, as well as provide training and education for local health care students who are unable to graduate due to the closure of schools in order to strengthen the resilience of the local health care system in Sierra Leone as well as the other EVD affected and high risk countries [10, 12]. Uganda has experienced six EVD outbreaks in 14 years, three of which appeared in 2011 and 2012 [13]. The first Ebola outbreak occurred in 2000 in Uganda's Northern District of Gulu and led to a total of 425 cases and 224 fatalities, making it the largest Ebola outbreak globally until the West African epidemic of 2014 [13–15]. Uganda has standing multi-sectoral and multidisciplinary task force committees on epidemics that include partners and NGOs at the national and district level [13]. The national task force is composed of experts (epidemiologists, laboratory scientists, communication experts, psychiatrists and psychologists, physicians, veterinarians, etc.) from the Ministry of Health, Ministry of Agriculture, Office of the Prime Minister and partners. The Partners include: WHO, CDC, UNICEF, AFENET, Uganda Red Cross, and MSF The NTF meets monthly; however it meets daily when there is an epidemic. Likewise, all the districts have task forces composed of the district political, civic, and health leadership as well as technical advisors from different partners working in the districts. Both the National and district task forces have subcommittees that are responsible for overseeing and implementing different components of epidemic response; the subcommittees include coordination, epidemiology and laboratory, case management, social mobilization, logistics, and psychosocial support and all have clear terms of reference. In addition, national rapid response teams and district rapid response teams are constituted immediately an epidemic is notified. They conduct investigations and support the establishment of an appropriate response in collaboration with the task forces. To reinforce response coordination at the district level, two or three senior officers are deployed from the national level to the district level to work with the district task force. During the inter-epidemic periods, monthly taskforce meetings are convened to review disease surveillance data and update epidemic preparedness and response plans. There is constant communication between district surveillance officers and the national level especially the division of disease surveillance in the ministry of health.

Over the years, the county's surveillance system has been strengthened. There are designated surveillance focal points at health sub-district, district, and regional levels, have been trained and facilitated to ensure timely detection, reporting, and investigation of priority diseases (including VHFs). These coupled with a good laboratory network within districts and at national level with built local capacities to conduct social mobilization, Infection control and case management have greatly contributed to Uganda's success in controlling previous Ebola/VHFs outbreaks. Uganda has been working with neighboring countries to strengthen the cross-border surveillance and management of epidemics. These response activities are implemented with close supervision by the National Task Force, the top leadership of the Ministry of Health, and the top Government leadership. Uganda is at a high risk of the Ebola transmission following the outbreak of Ebola in West Africa and the Democratic Republic of Congo. This is because of the high cross-border movements between these countries. Given Uganda's history of multiple Ebola outbreaks and looming risk of yet another EVD outbreak due to the ongoing outbreak in West Africa and neighbouring Democratic Republic of Congo, the Makerere University School of Public Health with support from the African Field Epidemiology Network (AFENET) organized an Ebola training exercise for the boarder districts. This paper intends to share lessons from this rapid Ebola preparedness and response capacity building exercise.

**Figure 1:**
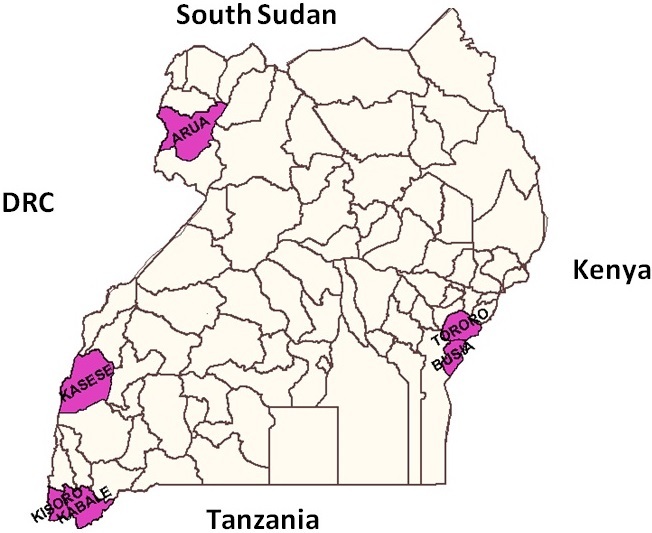
Map of Uganda showing the training sites (districts)

**Objectives for the Health workers'training:** the overall aim of the training was to empower health workers, immigration officers and other members of the multi-disciplinary multi-sectoral response teams in the border districts with knowledge and skills to conduct surveillance, investigate and respond effectively in the event that an Ebola Virus Disease (EVD) outbreak occurs. This was to set the country to the alert mode. The training was organised with several objectives depending on the group being trained and as for health care workers this included training and mentoring them on Ebola/VHF surveillance, preparedness and response; to guide the Health workers, district health teams in formulating District/HSD preparedness and reactivate district task force committees;to conduct awareness campaigns on Ebola case presentation & prevention in communities at border districts; and to provide logistical support to the districts for Ebola/VHF preparedness and response. The objectives of training for the the immigration officers/border staff were:; to orient health desk staffs and immigration officers on Ebola /VHF presentation, and surveillance and referral network and to train immigration staff and desk officers at Points of Entry on Public Health measures for preventing cross border transmission of VHFs (Figure 1).

## Methods

### Selection of the training sites

Six boarder districts were considered and these were Kisoro, Kasese, Kabale, Busia, Tororo and Arua. Sites with border posts that are used by many people from neighbouring countries were selected. These are in the districts of Arua, Kasese, Kisoro, Kabale, Tororo, Busia. The Democratic Republic of Congo has had numerous outbreaks and even the outbreaks that occurred in Uganda in Bundibugyo and Kibaale were in districts close to the border. There are also many refugees that have crossed from Congo into Uganda. There is therefore reason to include the districts of Kisoro, Kabale, Kasese and Arua in the border surveillance. Busia and Tororo are entry points for the main traffic from Kenya has an airport through which many passengers to Kenya and Uganda pass, entry points need to be put along this way to identify and support the efforts on this transit way.

### Trainee selection

The training included the District Health officers (DHO), health care workers mainly selected from Health Sub districts (HCII, HCIII, and HCIV) and the District hospitals and these included Medical Doctors, Nurses, and Laboratory Technicians among others. Immigration officers and boarder post personnel were selected from border posts. The border posts included Malaba (Tororo) Busia (Busia), Katuna (Kabale), Bunagana and Kyanika (Kisoro), Bwera-Mpondwe (Kasese) and Nvura Arua. These included immigration officers, and officers from different security organs like Police, Gombolola Security Officers (GISO's), Internal Security officers and External Security officers (ISO, and ESO), customs /trade agencies. Another key group of persons were the media who were invited and indeed participated so that their future reporting and communication to the population or communities they would do it professionally avoiding sensational information that would impact negatively on the real response efforts. The trainees were selected with support of the District Health Teams especially in close conjunction with the District Health officers and the surveillance officers. The DHO nominated the health workers and also linked up with other district departments to get a multidisciplinary team that would handle a possible ebola outbreak. The use of local technical leaders across different departments ensured that we got multidisciplinary teams that would all be sensitized so that in case an outbreak happens, as much as possible, the different departments can work together in sync. Each district provided health workers who were nurses, medical assistants, and Medical doctors. This ensured that both the public health and clinical care areas are organized together and would be able to work together in case of an outbreak.

### Training methodologies

The training was participatory using: group discussions, brainstorming; small group work, demonstrations, visual aids, role plays, case studies; and practical exercises. This tried to build on the experiences of these different professionals who were enhancing their expertise over and above their long experience in health care and surveillance. It also augured well with the adult training techniques for trainees of different disciplines. The training was done with mixed groups and sometimes with more specific groups aligned to the different disciplines [16]. The training lasted a week (five days) in each district to allow full commitment from the participants. A training of longer period would either not be attended by many people or some of the people would be off for some time and this would endanger the team spirit that the training was intended to achieve.

### Training Content

Because not much is known comprehensively on Ebola and what is known is not universal, careful preparations were done and the following were the main areas of focus, a) Overview of Integrated Disease Surveillance and Response(IDSR) implementation strategy in Uganda b) Framework for the International Health Regulations (IHR) Implementation in Uganda c) General Principles of Epidemic Preparedness d) Ebola Virus Disease (signs/symptoms, presentation and management) e) Ebola /VHFs (Alertness, Surveillance and Case definitions) f) Public Health Measures for control of Ebola/VHFs at major points of entry g) Action plans for setting up screening for Ebola/VHFs at border points h) Guidelines for screening travelers at Points of entry i) Reconstituting chlorine solutions for disinfection during Ebola outbreaks j) Standard Precautions for Infection Control in Health facilities and at Points of Entry k) Ebola/VHFs Specimen collection, processing, packaging & shipment l) Flow chart for Managing alerts, suspect cases & contacts m) Public Health Measures at major points of entry n) Guidelines on developing Epidemic Preparedness and response plans.

### Trainers

This training was carried out by Ugandan professionals drawn from MakSPH and Ministry of Health. The trainers were people who had been trained in field epidemiology or had been conducting surveillance programs and investigating outbreaks at district and Ministry of Health level. Some of the trainers had experience having been involved in the previous Ebola outbreaks in the country. Having a team with people with previous experience in outbreak investigations, having trained in field epidemiology and with experience of other outbreaks would help to equip the team with the knowledge necessary for the team, the expertise the experience of outbreak investigations.

## Results

### Training results/outcomes

**Figure 2:**
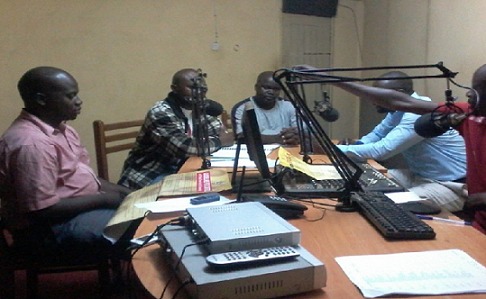
During the radio talk show at a Radio station in Tororo

Community engagement Radio talk show Three facilitators, and the district health educator were hosted on live call-in Health talked shows in both Tororo District In Kabale, the talk show was held at Radio West (FM 94.3) from 7PM to 9PM these being the pick time for the communities to be attending or listening to news and local informative programs. In Kisoro and Kasese districts radio the talk shows were held at voice of Muhabura 88.9 FM and Radio Messiah 97.5 FM respectively. These radio talk shows have in the past shown to be effective in mobilising the communities for a common goal [16]. The facilitators were given an opportunity talk about Ebola facts. Thereafter, the lines was then a live question and answer session with the listeners (Figure 2).

### Knowledge and skills gained

The participants in all the districts gained knowledge and skills that included; i. How to use PPE ii. Control of Ebola through infection control iii. Clinical presentation of Ebola iv. Diagnosis of Ebola and other Viral Haemeorrhagic Fevers v. Preparedness for Ebola Response vi. Materials needed for PPE vii. Standard precautions for infection control in health facilities viii. Meaning of Epidemic preparedness ix. Investigation and response to outbreaks x. Filter of rumors and case definitions xi. Action planning xii. Disease surveillance

### Topics most relevant and applicable to participants work

**Figure 3:**
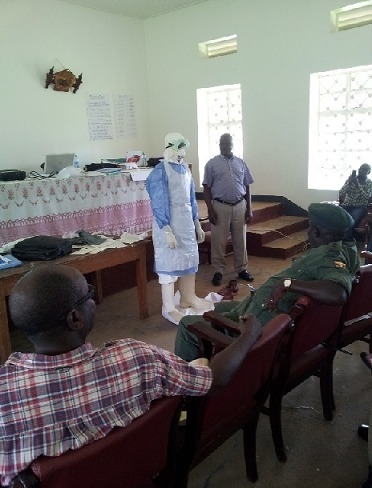
The demonstrating participant fully dressed up in the PPE

**Figure 4:**
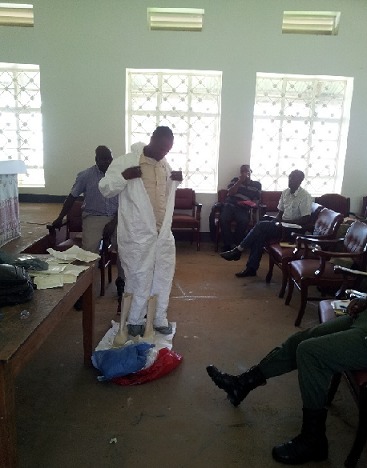
Participant demonstrating PPE removal process under the guidance of the facilitator

During the course of training it was noted that the topics that were highlighted as most relevant and applicable to participants’ work include:- a) Infection Prevention in Health care settings b) Developing Epidemic Preparedness and Response (EPR) Plans c) Management of Ebola d) Standard precautions for infection control e) Alert and surveillance, specimen collection, processing, packaging and shipping f) Mixing Jik/chlorine/bleach/Chlorex for elimination of germs g) Reconstituting Chlorine solution for infection control h) PPE dressing and undressing procedures i) Implementation EPR Plans j) Barrier nursing and care of patients k) Clinical management of Ebola l) Disease surveillance m) Hand washing n) Case definitions (Figure 3, Figure 4)

### General achievements of the trainings

**Figure 5:**
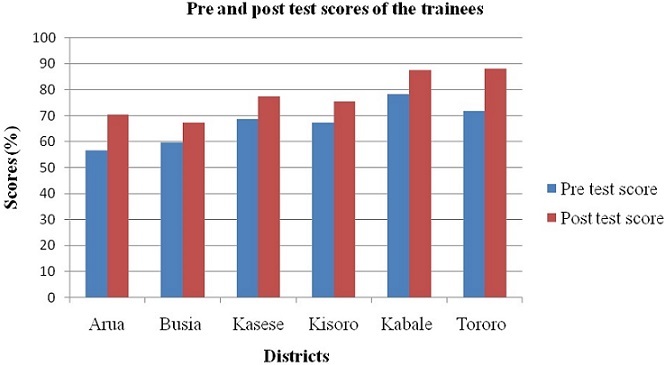
Pre and post test score of the trainees

**Figure 6:**
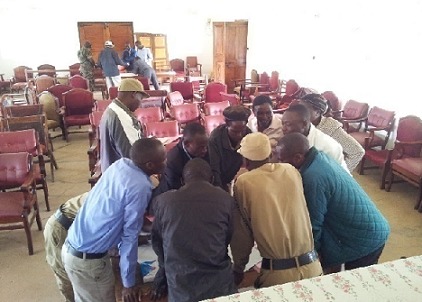
Participants developing epidemic prepardness and response plan

1) Over 210 health workers and 120 immigration staff/border officers and other multidisciplinary teams were trained in Ebola/VHFs surveillance, preparedness and response. Table 1 describes the social demographic characteristics of the trainees. *Uganda Peoples Defence Forces (i.e. Uganda's national army) (Table 1). 2) Our evaluation results demonstrated a gain in knowledge among trainees in all the districts as illustrated in Figure 5. 3) Communities in border districts were sensitized about Ebola and other VHFs through mass media, IEC materials, Radio talks shows and spot messages. Details of the distribution of the IEC materials are shown in Table 2. 4) All the six district received logical support in terms of PPE and other assorted item as part of the preparedness and response efforts as shown in Table 3 below. 5) District Epidemic Preparedness and Response plans were developed in all the six districts. The following key thematic areas were planned for: health worker training, laboratory strengthening, stockpiling of required material (PPE), seed funds, surveillance coordination and social mobilization. These will guide response actions in case of an outbreak in these districts: the capacity of screening at points of entry in all the district increased following the training. Currently plans are underway to create more screening points especially in Kasese district (Figure 6).

**Table 1:** Demographic characteristics of trainees

Characteristic	District					
	Arua(n,%)	Busia (n,%)	Kasese(n,%)	Kisoro(n,%)	Kabale (n,%)	Tororo(n,%)
**Sex**						
Male	39 (70.9)	34 (61.8)	45 (80.4)	39 (69.4)	49 (80.3)	30 (54.5)
Female	16 (29.1)	21 (38.2)	11 (19.6)	17 (30.4)	12 (19.7)	25 (45.5)
**Professional Background**						
Nurse/Midwife	16 (29.1)	20 (36.4)	18 (32.1)	16 (28.6)	17 (27.9)	16 (29.1)
Laboratory Technician	5 (9.1)	2 (3.6)	3 (5.4)	5 (8.9)	2 (3.3)	5 (9.1)
Health assistants/DHT	4 (7.3)	5 (9.1)	4 (7.1)	3 (5.4)	6 (9.8)	1 (1.8)
Clinical officer	6 (10.9)	5 (9.1)	11 (19.6)	8 (14.3)	10 (16.4)	9 (16.4)
Medical officers	4 (7.3)	3 (5.4)	0 (0)	4 (7.1)	5 (8.2)	4 (7.3)
Immigration officers	8 (14.6)	5 (9.1)	3 (5.4)	2 (3.6)	1 (1.6)	2 (3.6)
Customs officers	3 (5.4)	6 (10.9)	3 (5.4)	4 (7.1)	3 (4.9)	3 (5.5)
Police officers	5 (9.1)	5 (9.1)	6 (10.7)	8 (14.3)	6 (9.8)	10 (18.2)
UPDF*	2 (3.6)	3 (5.4)	2 (3.6)	0 (0)	5 (8.2)	0 (0)
Other security personnel	1(1.8)	0 (0)	5 (8.9)	5 (8.9)	2 (3.3)	4 (7.3)
Media	1(1.8)	1 (1.8)	1 (1.8)	1 (1.8)	4 (6.6)	1 (1.8)

UPDF, Uganda People's Defence Forces, other security personnel include border and sub county security officer. Nurses/midwives include both registered and enrolled nurses and midwives.

**Table 2:** Information, Education and communication (IEC) support to Districts

Item	Units per District	Numbers of District	Quantity for six districts
**Mass Media**			
Radio spot messages on Ebola/VHFs	150	6	900
Radio talk shows on Ebola/VHFs	1	6	6
**Posters**			
A1 (Full colour, Art paper)Guidelines for Control of Ebola fever in the Community	200	6	1,200
A2 (full colour, Art paper)Guidelines for Control of Ebola fever in the Community)	800	6	4,800
Leaflets/fliers(full colour Art paper)Facts About Ebola	800	6	4,800
A4 sides (full colour, Art paperFacts About Ebola	200	6	1,200

**Table 3:** Distribution of PPEs and Assorted items for infection prevention and control per district

PPEs/Assorted Items	Units per district	Number of districts	Total Quantity of items for six districts
Spray Pumps	10	6	60
Ordinary Gum boots (white)	50	6	300
Dust bins	6	6	36
Alcohol based hand sanitizers 60MLS	60	6	360
Jik (1 litre)	60	6	360
Water proof heavy duty Aprons -plastic- re-usable	30	6	180
Heavy duty gloves	30	6	180
Bed sheets (60 x 90) 2 sheets	22	6	132
Medical masks	50	6	300
Goggles/face shields	50	6	300
Medical cover overalls /Gowns	50	6	300

## Discussion

Previous outbreaks have largely been contained without multiple transmission to other parts of the world and often only affected a few hundred people. The current outbreak with such a world wide spread mandates that all countries including the U.S. medical and public health systems were called upon to prepare adequately for Surveillance, Preparedness and Response by training the health workers and many other multidisciplinary teams [1]. The use of personal protective equipment (PPE) and adoption personal protective behaviours that can be used to prevent its spread and reduce exposure are key to the global preparedness and response efforts [17]. There is a lot of research that has been done in laboratory setting but more work will be needed in or collected from real world settings (e.g., outside the laboratory) to inform the world how best to respond [18, 19]. This need should take into account the cultural behavior, social and psychological impact of the disease and indeed the role of Governments in the control of Ebola and related diseases that affect communities. The handling of the dead and potentially infected contaminants remain one of the biggest challenges in the arrest of the spread and this poses one of the greatest challenges to modern Public health [20]. There is need to provide for improved health services and systems as a whole but this is not achievable in such a short time given that the disease attacked the most vulnerable countries in terms of the health care systems.

## Conclusion

1) Site selection for the training should put into consideration possible engagement with an Ebola outbreak. In such a situation, the participants would see what they are trained in as relevant and applicable. 2) Trainee selection: The trainees should be of multidisciplinary nature since the Ebola outbreak would need different sectors to work together and when these have received a similar training and more so together, then they would be able to handle such an emergency more effectively that if they never had a similar training. 3) Training content: the training content should be focused to the issue at hand, build on the knowledge these different teams have and demonstrate the different areas where each member of the team is to play a part. 4) Trainers: Trainers who are experts in field epidemiology, have had hands on experience in conducting outbreaks and more so similar outbreaks are an essential resource for a training of this nature. They would give live examples and stories that trainees can easily identify with. 5) Training methods: participatory training methods work well for teams that are composed of people from different professions, that have people with a lot of experience already and are adults. This helped to drive the message home, keep the commitment of the participants and build on the knowledge base that the participants had. By so doing, the participants so the training as part of their work and not something outside their mandate. 6) Community engagement: Radio talk shows and especially putting in a questions and answer session helps the community to engage with the experts, helps the trainers to identify the grey areas that need to be clarified but also built a community engagement with the health workers. The district health team would build on what has already been established. 7) The success of training could be replicated in other places that are potentially areas where Ebola would enter the country but when adapted, the training can also mobilize the health workers and different teams in local communities to address an Ebola outbreak.
